# Supplementary material for: Testing for reviewer anchoring in peer review: A randomized controlled trial
Source: PLoS One. 2024 Nov 18;19(11):e0301111. doi: 10.1371/journal.pone.0301111 (PMC11573134; doi:10.1371/journal.pone.0301111)
Supplement: S1 Appendix — (PDF) [file pone.0301111.s001.pdf]

S1 Appendix

Deception and revision process. In this appendix, we first outline the full deception and revision procedure starting from when experimental participants finish their initial review, and ending after they submit their revised review. Then, we list some common deviations to the expected procedure that happened in practice, as well as how we addressed them to return to the experimental procedure.

In the experimental procedure, when the participants view the paper on their browser, they see a frozen version of the GIF figure that contains the paper's main evaluation result. The GIF result (pictured in Figure 1) still fits into the context of the paper text due to its timeline structure, but its result is substantially weaker than the result depicted in the full GIF animation. Furthermore, there is no mention of the specific numerical values of the main result anywhere outside the GIF figure, and the text surrounding the figure is also intentionally vague to allow for both versions to avoid any inconsistencies between figure and text.

Review questions are situated on a google form separate from the webpage. The first page consists of all the traditional review questions, while the second page contains background questions and comments, such as recording the institution and year of the participant. This second page also includes the question, "Please comment on the use of animated figures. (If you did not see this form of media, please answer 'N/A'.)". When the experimental group participants encounter this question, as they did not see any animated figures, they should answer "N/A". Once they complete their review, the experimenter verbally announces that they are taking a quick look over their submitted review. Meanwhile, the experimenter is actually changing the contents of the webpage that hosts the paper to incorporate the working GIF instead. The experimenter then deceives the participant by acting confused about their "N/A" response to the animated figures question. They state that the participant should have seen an animated figure, and ask them if they could reload the page or try a different browser.

Once the participant loads the paper again, they see the animated GIF and notify the experimenter that they had not previously seen the animation. The experimenter then asks them to edit their review based on the figure change, and provide them with the same google form link so that they can edit their response. Prior to their new response being submitted, the experimenter also downloads the participant's original response.

We chose to have reviewers revise their initial responses because this parallels the situation in which reviewers revise their ratings after being given rebuttals. Often, conferences will have a reviewer's initial review available as either a reference or to directly edit over, which we mirrored in our experimental setup. We also ensured that reviewers are informed that they could edit any part of their review, not just the comment regarding animated media.

Deviations to the expected procedure and prepared solutions. In this section, we describe the responses we had in place in case any parts of the experiment did not go as according to the procedure. These originated from both our initial planning and our experiences in the pilot study.

One common mistake that experimental participants made was that they mistakenly believed that the static figure shown initially was the "animated figure" referenced in the review form question, despite it not being animated. Consequently,

they answered the animated figures question incorrectly by commenting on the static figure instead. This disrupted our attempts to notify them that they saw the wrong figure, which was normally done through this question. To address this, when we identified that participants were mistaken in this way, we instead asked them a follow-up question to clarify their answer to the animated figures question, such as "Could you elaborate a bit more on your answer?". Then, when the participant explained their answer, the experimenter could act confused as the figure they described would not have an animated component, thereby transitioning back to the experimenter "noticing" that the participant had not seen an animated figure, and asking the participant to reload the page.

Another somewhat frequent question from experimental group participants was whether they were supposed to see an animated figure. Here, we could not give them a yes or no answer, as "yes" would reveal that there was a mistake prematurely, while "no" would contradict ourselves later on. In this situation, we instead pretended that the experiment was double blind, stating that we also did not know if they were supposed to see an animated figure until they submitted their review. Then, after their reviews were submitted, we notified them that they were actually supposed to see an animated figure. To keep our control and experimental conditions consistent, we attempted to answer all questions from participants identically regardless of which group they were in.
