# Supplementary material for: Testing for reviewer anchoring in peer review: A randomized controlled trial
Source: PLoS One. 2024 Nov 18;19(11):e0301111. doi: 10.1371/journal.pone.0301111 (PMC11573134; doi:10.1371/journal.pone.0301111)
Supplement: S2 Appendix — (PDF) [file pone.0301111.s002.pdf]

S2 Appendix

Power analysis. To determine the target number of participants for our study, we performed a power analysis for our original pre-registered significance test. In the power analysis, we assumed that the control and revised Overall scores were distributed normally with two corresponding fixed variances. Since participants review the same paper, the variances were chosen by randomly sampling reviewer score variances across individual papers in ICLR 2022[1], with different values for each trial of the permutation test. We chose the ICLR 2022 conference due to its proximity to the fake paper's topic as a machine learning conference, as well as its open-source review score data that we could sample from. Overall scores in ICLR 2022 were also based on a 10-point scale, with an average of 3.85 reviewers per paper. We chose to sample two separate variance values for the control and revised scores as participants in different groups might have had different perceptions of the paper.

Based on our analysis, we targeted a minimum of 100 participants, since this corresponded to an estimate that we would be able to detect a 0.25 difference in means between the control and revised scores ($\alpha = 0.05, \beta = 0.2$).

However, the variances we obtained during data collection were much higher than the estimate (see Table 8). In hindsight, we note two limitations of our initial variance estimate:

1. The scores we used were the post-rebuttal scores, as pre-rebuttal scores were not openly available. In reality, it may be the case that post-rebuttal scores are closer than pre-rebuttal scores due to rebuttals or reviewers being influenced by other reviewers' reviews (this behavior is discussed in Sections 2.1 and 2.2).

2. The participants in our study may have had less homogeneous backgrounds than the typical set of reviewers for a paper. Though our participants were largely of the same age group and social environment, they came from many different subfields of computer science, and thus may have had differing impressions about the standards for an 'accept' submission. This may also have contributed to an increased variance in scores.

Table 8. Average intra-paper Overall score variance from the ICLR 2022 dataset, as well as the variances of our initial, revised, and control Overall scores.

	ICLR-22	Initial	Revised	Control
Variance	1.53	2.69	2.53	2.00

All scores are on a 10-point scale.

[1]available at https://docs.google.com/spreadsheets/d/1Fdop2Byzhxx5gKI4YZXqq_L-akDG8GwdGMqb_4IbS38/edit?gid=232519225#gid=232519225
