# Supplementary material for: Testing for reviewer anchoring in peer review: A randomized controlled trial
Source: PLoS One. 2024 Nov 18;19(11):e0301111. doi: 10.1371/journal.pone.0301111 (PMC11573134; doi:10.1371/journal.pone.0301111)
Supplement: S3 Appendix — (PDF) [file pone.0301111.s003.pdf]

S3 Appendix

Participant recruitment. We had four requirements for participants to join the study. First, participants were required to be either a current PhD student or have already obtained a PhD. Second, participants were required to have at least one publication in a computer science-related field within the last 5 years, up to the date of the study. Third, participants were required to over the age of 18. And finally, participants were required to be currently residing in the United States. Given these requirements, our participants were likely to be either current or future reviewers at computer science conferences: 33% of reviewers at the NeurIPS 2016 conference were PhD students [4].

We recruited participants through physical posters, emails to PhD-student mailing lists, social media posts, announcements to students in PhD-level courses, door-to-door recruitment at PhD offices, as well as word-of-mouth. These methods were performed to varying degrees (depending on physical limitations) at Carnegie Mellon University and 8 other research universities. Participants were given a QR code or link to a sign-up calendar, where they could select their own 30-minute meeting timeslot with the experimenter.
