# Supplementary material for: Testing for reviewer anchoring in peer review: A randomized controlled trial
Source: PLoS One. 2024 Nov 18;19(11):e0301111. doi: 10.1371/journal.pone.0301111 (PMC11573134; doi:10.1371/journal.pone.0301111)
Supplement: Attachment — Submitted filename: Reviewer Letter for Anchoring Submission - Google Docs.pdf [file pone.0301111.s004.pdf]

Response to Reviewers:

Reviewer #1:
Thank you for your careful review of our manuscript. We have made changes to the paper according to your following suggestions:
- page 1, second line from bottom: replace "change" with "adapt"
- page 11, line under Section 4 - delte this unnecessary sentence "In this section..."
You can find these changes in the “track changes” version of the revision.

Reviewer #2:
Thank you for your detailed comments and suggestions for our manuscript. In particular, we appreciate your careful consideration the various facets of our experiment design. We would like to briefly discuss your concerns regarding our paper:

1. The experimental setup of the article has certain limitations, such as the construction of only one fake paper.

Response:
We agree with the reviewer that this is indeed a limitation to our work. We have added a paragraph in the discussion specifically discussing this limitation. We reproduce the paragraph here for the reviewer's convenience:

“Another limitation of our work is that we run our experiment with only one paper, which could lead to our findings to be less generalizable. There is precedence of research involving reviewers reviewing fake papers, and in each of these only 1 to 3 papers are constructed [1–5]. Due to the high sample size determined from the power analysis (see S2 Appendix) and the limited pool of eligible participants (see S3 Appendix), we chose to have one paper to reduce the sample size needed in order to test for statistical significance, as having multiple papers would require an additional random effect to be modeled. Future work may also include papers from multiple domains to bolster the generalizability of the study.”

2. The data provided by the authors is somewhat scant, although they have discussed the limitations.

Response:
We provide the full anonymized numerical responses and participant institution and year in the data folder of https://github.com/theryanl/ReviewerAnchoring. Our IRB approval and the consent of participants do not permit us to include participant comments in the dataset. This was due to potential concerns of anonymity breach (e.g., “Since I work in the neighboring field of xxx, …”). The participant pool, mainly CS or CS-related field PhDs, as well as their institution and year are provided. If additional information regarding their area of study can be inferred from their responses, this may cause certain participants’ identities to be identifiable.

3. The gap between the authors' experiments and the real world is considerable (e.g., peer effect, peer pressure, rebuttal skills, rebuttal politeness), making it challenging to assure the validity of the findings on real-world data. While considering these factors is challenging, if the authors claim to study the anchoring phenomenon in peer reviews, then these factors cannot be overlooked.

Response:
As you mention, there are many other peer effects in the process of rebuttals. The design of this experiment deliberately avoids adding these additional effects in order to isolate the effect we wish to understand – anchoring in its base form (as defined by [6]) without social or author effects. We believe this base form is important to analyze, as an existence of anchoring in this setting would mean that the re-reviewing paradigm itself causes anchoring bias to happen, meaning that altering social pressures and other effects would be insufficient to avoid bias. This setting is also consistent with anchoring bias studies in psychology which did not contain external social pressures or author skills or politeness [6].

In this case, adding other peer effects or author skills and politeness can confound any observations that we make. These factors are orthogonal to anchoring, and our objective in the experiment design is to single out anchoring and avoid these other confounders. This is actually a flaw with the study by Gao et al. you cited –

the paper claims "peer pressure" as a reason for their observation, but their study does not isolate such effects and hence the claim can be confounded by various factors such as program chair instructions to arrive at a consensus. Our study, in contrast, asks a specific question (of anchoring), and the experiment design deliberately and carefully avoids other such confounders in order to answer this specific question. By coming to a conclusion on just anchoring, this also allows us to better evaluate the existence of other effects in future studies, and possibly help inform the design of future peer review paradigms.

4. Please revise some arxiv paper reference into their proceedings versions.

As you suggested, we have made changes to the references, replacing arxiv paper references to their proceedings versions. They should be up-to-date with Google Scholar as of 2/9/2024.

[1] W. G. Baxt et al. “Who reviews the reviewers? Feasibility of using a fictitious manuscript to evaluate peer reviewer performance”. In: Annals of emergency medicine (1998).
[2] G. B. Emerson et al. “Testing for the presence of positive-outcome bias in peer review: a randomized controlled trial”. In: Archives of internal medicine (2010).
[3] F. Godlee, C. R. Gale, and C. N. Martyn. “Effect on the quality of peer review of blinding reviewers and asking them to sign their reports: a randomized controlled trial”. In: JAMA (1998).
[4] S. Schroter et al. “Effects of training on quality of peer review: randomised controlled trial”. In: BMJ (2004).
[5] S. Schroter et al. “What errors do peer reviewers detect, and does training improve their ability to detect them?” In: Journal of the Royal Society of Medicine (2008).
[6] A. Tversky and D. Kahneman. “Judgment under uncertainty: Heuristics and biases.” In: Science (1974).
